# Effects of gestational weight gain on emotional and behavioral problems in children: Results from the CLaB study

**DOI:** 10.1371/journal.pone.0329762

**Published:** 2025-08-19

**Authors:** Caroline de Barros Gomes, Cristina Maria Garcia de Lima Parada, Ana Beatriz Henrique Parenti, Giovana Canela Spadotto, Michelly da Silva Alves, Flávia Helena Pereira Padovani, José Eduardo Corrente, Maria Antonieta de Barros Leite Carvalhaes

**Affiliations:** 1 Post-graduate program in Public Health, São Paulo State University (UNESP), Medical School, Botucatu,; 2 Post-graduate program in Nursing, São Paulo State University (UNESP), Medical School, Botucatu,; 3 Research Support Office, São Paulo State University (UNESP), Medical School, Botucatu; Chinese Academy of Medical Sciences and Peking Union Medical College, CHINA

## Abstract

**Background:**

Gestational weight gain (GWG) may influence child neurodevelopment, with potential effects on emotional and behavioral outcomes. This study investigates the relationship between GWG and behavioral problems in children aged 7–8 years.

**Methods:**

This cohort study used data from the Botucatu Infant Cohort. The first wave (2015–2016) included 656 newborns and their mothers, with maternal GWG classified as insufficient, adequate, or excessive according to National Academy of Medicine guidelines. The second wave (2023–2024) assessed 394 children aged 7–8 years using the Strengths and Difficulties Questionnaire (SDQ) to identify emotional and behavioral problems (scores ≥17). We used Poisson regression adjusted for confounders to test whether GWG adequacy (insufficient, adequate, or excessive) was associated with total, internalizing (≥8), and externalizing (≥11) behavioral problems.

**Results:**

Among 309 children with complete data, 36.2% presented scores indicative of behavioral problems on the SDQ; 37.5% showed internalizing problems, and 27.5% exhibited externalizing problems. When adequate GWG was the reference category, prevalence ratios varied but did not reach statistical significance. However, when excessive GWG was the reference, children of mothers with insufficient weight gain had a higher prevalence of behavioral problems (PR = 1.48; 95% CI: 1.02–2.13). In contrast, compared to children whose mothers had adequate weight gain, those whose mothers gained excessive weight had fewer internalizing problems (PR = 0.66; 95% CI: 0.47–0.94).

**Conclusion:**

The prevalence of behavioral problems assessed by the SDQ was very high, highlighting a significant issue in the study context. However, our hypothesis that GWG outside recommended ranges could influence behavioral problems in children aged 7–8 years was only partially confirmed. Insufficient GWG was associated with overall behavioral problems (SDQ > 17), while excessive GWG appeared protective against internalizing problems. No association was found between GWG and externalizing problems.

## Introduction

Epigenetic studies have highlighted the importance of the gestational period for physical and mental health throughout life. Adverse exposures during pregnancy and early childhood have shown lasting impacts on the health of both mother and child [1].

Inadequate gestational weight gain (GWG) – either excessive or insufficient – is a marker of adverse intrauterine conditions, with negative impacts on the health of both mother and baby [2]. These repercussions extend beyond the gestational period and birth, encompassing impacts on the development, cognition, and mental health of offspring [3,4]. In this context, it is recognized that gestational weight gain affects neurodevelopment [5], and behavioral repercussions are also likely, often assessed using the Strengths and Difficulties Questionnaire (SDQ) [6].

A historical cohort study in Japan found an inverted J-shaped relationship between gestational weight gain and total SDQ scores in children aged 6–7 years. Gestational weight gain below 10 kg increased the risk of clinically relevant outcomes in the total score, while values above this threshold showed no significant effect. Quintile analysis revealed that the first quintile (<7 kg) increased the risk of behavioral problems, while the fifth quintile (>14 kg) reduced the likelihood of problems in the prosocial domain [7].

Using data from the Avon Longitudinal Study of Parents and Children (ALSPAC), associations between gestational weight gain and child behavior from ages 3–16 years were analyzed in over 6,800 mother-child pairs at age 3 and 3,925 pairs at age 16. The study found no evidence of association, except for a reduced risk of prosocial behavioral problems at ages 9 and 11 in cases of excessive gestational weight gain [8].

Another study in this same line investigated the association between GWG and behavioral problems in children aged 6–7 years, using data from two European birth cohorts (MEFAB in the Netherlands and Rhea in Greece). Behavioral problems were assessed in two main domains: internalizing, which includes emotional difficulties like anxiety and withdrawal, and externalizing, which involves outward behaviors such as aggression and hyperactivity. Among mothers with pre-pregnancy overweight or obesity, higher GWG was associated with higher scores in both domains, whereas among mothers with normal BMI, the associations were inconsistent [9]. In contrast, findings from the Canadian APrON cohort, which assessed children aged 3–5 years using the BASC-2 instrument, found no significant associations between maternal pre-pregnancy BMI or GWG and internalizing or externalizing behaviors [10].

The relationships between pre-gestational nutritional status, gestational weight gain, and child behavior, as well as the diversity of methods and findings in previous studies, highlight the need for further investigation. The high prevalence of behavioral problems in children [11] and the global frequency of excessive and insufficient gestational weight gain [12] justify exploring these factors as modifiable determinants. Investigating these associations is crucial, as gestational weight gain can be modified through interventions in health services [13]. This study aimed to examine the association between gestational weight gain and behavioral problems in children aged 7–8 years.

## Methods

### Study design and data source

This paper analyzes data obtained from the Botucatu Infant Cohort Study (CLaB) during its first and second waves. The first wave included newborns and their mothers, collecting maternal information on pregnancy, childbirth, the postpartum period, and up to the first year postpartum. For infants, growth, development, feeding, morbidity, and use of health services were investigated until they reached one year of age. Socioeconomic and demographic data of the families were also collected. Among the maternal data, gestational weight gain, the exposure variable analyzed in this study, is highlighted.

In the second wave, conducted in 2023 and 2024, the cohort children, now aged 7–8 years, were reassessed for health aspects, morbidity, nutritional status, diet, mental health status, and behavior. The presence of behavioral problems constitutes the outcome variable under study.

### Study location

The city of Botucatu, where the cohort resides, is in the south-central region of São Paulo State, Brazil. At the time of the first wave, it had a population of 138,000 inhabitants, which increased to approximately 145,000 by 2022, according to the latest. It is a city with a Human Development Index (HDI) of 0.800, higher than the Brazilian average, with an economy predominantly based on the service sector.

### Study population

During recruitment for the formation of the CLaB cohort, newborns residing in the urban area of the municipality who attended neonatal screening tests provided by the Brazilian Unified Health System were included. These tests were scheduled by maternity hospitals and conducted in a centralized public unit, which covered approximately 90% of all newborns. To be included, mothers needed cognitive, verbal, and auditory abilities sufficient to respond to in-person and telephone interviews.

The original cohort consisted of 656 newborns and 650 mothers. At the end of the 12-month follow-up, 585 infants were evaluated, with losses (10.8%) primarily due to unavailability for interviews.

In the second wave, 394 of the 585 infants who completed the 12-month follow-up were located. After completeness and consistency analysis, valid data on maternal gestational weight gain, pre-gestational maternal nutritional status (from the first wave), and child behavior (second wave) were obtained for 309 children, forming the sample for this study.

With this sample, a 30% frequency of children with a total SDQ score >17, and a 20%-point difference between children whose mothers had insufficient and excessive weight gain, the study power is 0.9849 (98%).

### Data collection and study variables

The first phase of the CLaB study took place from July 27, 2015, to February 24, 2017, and involved face-to-face interviews when the child was less than one month old and at 3, 6, 9, and 12 months, as well as telephone interviews at 2 and 4 months. These were conducted by a trained and supervised team, guided by the field researcher. Data was recorded on paper forms, double-entered to identify errors, and approximately 5% of interviews were partially repeated to confirm or correct implausible data. Further details can be found in previous publications [14].

The second phase took place from January 25, 2023, to July 25, 2024, and included both face-to-face and telephone interviews. The first in-person interview, conducted at home, collected updated sociodemographic data on the mother, father, and family, including morbidity and healthcare history. Data were recorded on paper, entered using Survey Monkey® software, and exported to Microsoft Excel for consistency checks before being imported into statistical software. Two telephone interviews collected detailed data on dietary intake and the child’s participation in sports and artistic activities, among other data excluded from this analysis. In both phases of the study, the interviewers were external professionals who had no prior contact with the children or their families.

Gestational weight gain, the exposure variable, was calculated using data from the prenatal card. Pre-gestational weight was based on the card’s recorded weight (typically self-reported) or the first trimester weight if discrepancies of two kilograms or more existed [15]. Gestational weight gain was calculated by subtracting pre-gestational weight from the weight measured at the last prenatal visit, adjusted for the child’s gestational age at birth, as per the formula below:


$$Total\;GWG=\frac{WLPN-PPW}{\begin{array}{c} GALPN \end{array}}\times GAB\mathrm{\backslash ]}$$

WLPN = weight at last prenatal visit

PPW = pre-pregnancy weight

GAB = gestational age at last prenatal visit

GALPN = gestational age at birth

Subsequently, gestational weight gain was categorized as insufficient, adequate, or excessive based on the 2009 National Academy of Medicine guidelines, which define desirable weight gain according to pre-gestational nutritional status: 12.5–18.0 kg for underweight women, 11.5–16.0 kg for normal weight, 7.0–11.5 kg for overweight, and 5.0–9.0 kg for obesity [16].

Emotional/behavioral problems in the child were identified using the Strengths and Difficulties Questionnaire (SDQ), Parent Version [6]. The SDQ is an open-access tool with 25 items (www.sdqinfo.com), developed to screen for mental health problems in children aged 4–17 years over the preceding six months. The Brazilian version was validated by Fleitlich-Bilyk and Goodman [17]. Its 25 items are distributed across five scales: emotional symptoms, conduct problems, hyperactivity/inattention, peer problems, and prosocial behavior. The total score is calculated by summing the four difficulty subscales, excluding prosocial behavior, which measures resources rather than problems. Scores ranging from 17 to 40 indicated emotional and behavioral problems [17].

Partial scores for the four difficulty domains used the following abnormality cutoff points: emotional symptoms (5–10 points), conduct problems (4–10), hyperactivity (7–10), peer problems (4–10), and prosocial behavior (0–4) [6].

Responses to the SDQ were grouped to identify two types of behavioral problems. Internalizing problems comprise two subscales (10 items): emotional symptoms (somatic complaints, worries, unhappiness, nervousness, and fears) and peer problems (being solitary, lacking good friends, being unpopular, bullied, and preferring adult company), with scores ranging from 0 to 20. Externalizing problems also comprise two subscales (10 items): hyperactivity/inattention (restlessness, fidgetiness, distractibility, lack of reflection, and inattention) and conduct problems (temper outbursts, disobedience, fighting, lying, and stealing), also with scores ranging from 0 to 20 [6,18]. We then investigated the association between GWG and the presence of internalizing and externalizing behavioral problems, using cutoff points of 8 and 11, respectively [18].

### Statistical analyses

Descriptive data from the SDQ questionnaire results, both for the total score and for the scores in the different subdomains, were presented in terms of mean values and respective standard deviations (SD), medians, interquartile ranges, and minimum and maximum values. The comparison of these values according to the adequacy of maternal gestational weight gain was then conducted using the Kruskal–Wallis test. We also compared the frequencies of children with and without behavioral problems based on the total SDQ score according to the covariates, with differences tested using Pearson’s chi-square or Fisher’s exact test.

To assess the association between the classification of gestational weight gain (insufficient, adequate, or excessive) and the presence of behavioral problems (SDQ ≥ 17), we performed Poisson regression analyses with robust variance, both bivariate and multivariable, calculating prevalence ratios and their respective 95% confidence intervals. Based on preliminary analyses that indicated a negative linear relationship between GWG (as a continuous variable) and the SDQ score, we chose to conduct the analyses using the categorized classification of weight gain, with two different reference categories: adequate and excessive.

**Fig 1:**
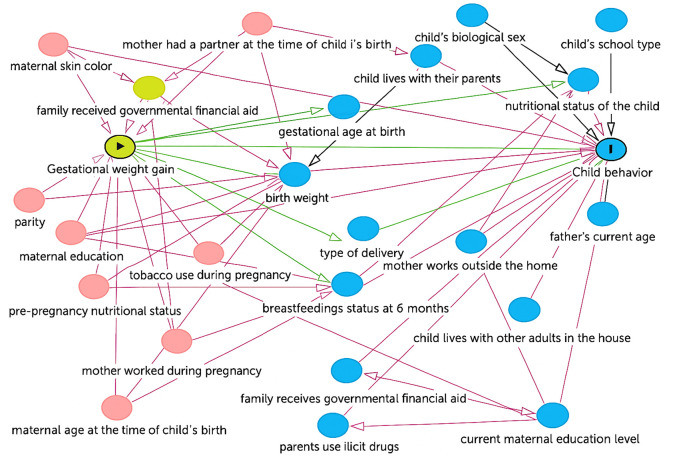
Directed Acyclic Graph between gestational weight gain and child behavior.

The selection of potential confounders to be included in the multivariable analyses was based on the literature and by constructing a Directed Acyclic Graph (DAG) using the Dagitty software (https://www.dagitty.net/) (Fig 1), which indicated the need to include the following factors in the multivariable model: smoking during pregnancy, living with a partner during pregnancy, maternal skin color, education, pre-gestational nutritional status, maternal age, parity, and maternal employment during pregnancy. The child’s sex was also considered, although it did not influence gestational weight gain, due to the recognized difference between girls and boys in terms of behavioral problems [19,20]. Similar analyses (bivariate and multivariable) were conducted for each of the five SDQ domains: internalizing and externalizing problems.

All analyses were performed using the Statistical Package for Social Sciences, v.29.0, considering p < 0.05 as the level of statistical significance.

### Ethical aspects

The first phase of the CLaB study was evaluated and approved by the Research Ethics Committee of the Botucatu Medical School, São Paulo State University-UNESP, under CAAE: 37337314.3.0000.5411. The second phase was also approved by the same Committee (CAAE: 57893322.7.0000.5411).

In accordance with national and international ethical guidelines for research involving human subjects, written informed consent was obtained from all mothers or legal guardians of the participating children. Consent was formally documented through the signing of a Free and Informed Consent Form prior to the initiation of both data collection phases.

## Results

Among the 309 children in the sample of this study, the average SDQ score was 13.9 (SD 7.3) points, with a minimum score of 0 and a maximum score of 34. A total of 112 children (36.2%) had scores indicative of behavioral problems (SDQ ≥ 17 points). Regarding the subscales, the average score was 4.6 (SD 3.1) on the hyperactivity scale, 4.3 (SD 2.7) on the emotional symptoms scale, 2.8 (SD 2.5) on the conduct problems scale, 2.2 (SD 1.9) on the peer problems scale, and 9.1 (SD 1.4) on the prosocial behavior scale. On the emotional symptoms scale, 46.3% had scores indicative of abnormality, while this percentage was 33.7% on the conduct problems scale, 30.4% on the hyperactivity scale, and 9.7% on the peer problems scale. Only 2.3% had abnormal scores on the prosocial behavior scale. The prevalence of internalizing problems was 37.5%, while that of externalizing problems was 27.5% (data not presented in a table).

In Table 1, the characteristics of the studied population are shown, both overall and separately for children above and below the threshold indicative of behavioral problems (total score). Most mothers were between 20 and 34 years of age (68.9%), with a high frequency of first-time mothers (45.6%) and those with intermediate education (66.3%). The distribution of GWG adequacy showed higher rates of excessive gain (39.2%), followed by adequate gain (33.3%) and insufficient gain (27.5%). Gestational weight gain, maternal skin color, living with a partner, and maternal employment outside the home at the time of the child’s birth, as well as maternal education level and birth location, showed differences between children with scores above or below 17 points. Among the variables regarding the child at 7/8 years of age, only mental health follow-up showed a statistically significant difference. Variables related to the mother or family in the second wave did not show any association with the SDQ score classification at 7/8 years of age.

**Table 1:** Characteristics of mothers and children according to SDQ score. CLaB Study, Botucatu, Brazil, 2016-2024 (n = 309).

Characteristics	Total N (%)	SDQ ≥ 17 points N (%)	SDQ < 17 points N (%)	p-value
**Maternal Age at birth**				0.108
20–34 years	213 (68.9)	80 (71.4)	133 (67.5)	
18–19 years	46 (14.9)	20 (17.9)	26 (13.2)	
35 years older	50 (16.2)	12 (10.7)	38 (19.3)	
**Maternal skin color**				0.026
White/ Yellow	1 (58.6)	59 (52.7)	2 (2.6)	
Black	26 (8.4)	6 (5.4)	20 (26.0)	
Mixed race	102 (33.0)	47 (42.0)	55 (71.4)	
**Mother had a partner at the time of the child’s birth**				0.002
Yes	266 (86.4)	90 (80.4)	176 (89.8)	
No	42 (13.6)	22 (19.6)	20 (10.2)	
**Maternal employment status at childbirth**				0.036
Yes	176 (57.0)	55 (49.1)	121 (61.4)	
No	133 (43.0)	57 (50.9)	76 (38.6)	
**Maternal education at birth (in completed years of schooling)**				0.002
≥ 12	50 (16.2)	25 (12.7)	25 (22.3)	
9–11	205 (66.3)	145 (73.6)	60 (53.6)	
< 8	54 (17.5)	27 (13.7)	27 (24.1)	
**Parity**				0.851
Primiparous	141 (45.6)	53 (47.3)	88 (44.7)	
2–3 children	146 (47.2)	52 (46.4)	94 (47.7)	
4 or more children	22 (7.1)	7 (6.3)	15 (7.6)	
**Maternal smoking during pregnancy**				0.075
Yes	29 (10.2)	15 (14.4)	14 (7.8)	
No	255 (89.8)	89 (85.6)	166 (92.2)	
**Family received governmental financial aid at the time of the child’s birth**				0.354
Yes	27 (8.7)	12 (10.7)	15 (7.6)	
No	282 (91.3)	100 (89.3)	182 (92.4)	
**Pre-pregnancy nutritional status**				0.153
Normal weight	142 (46)	51 (45.5)	91 (46.2)	
Underweight	10 (3.2)	7 (6.3)	3 (1.5)	
Overweight	87 (28.2)	23 (20.5)	58 (29.5)	
Obesity	70 (22.7)	25 (22.3)	45 (22.9)	
**Gestational weight gain (according to IOM and NRC. 2009 guidelines)**				0.045
Adequate	103 (33.3)	35 (34.0)	68 (66.0)	
Insufficient	85 (27.5)	40 (47.1)	45 (52.9)	
Excessive	121 (39.2)	37 (30.6)	84 (69.4)	
**Child’s place of birth**				
Public healthcare system	218 (70.6)	89 (78.8)	129 (65.5)	0.010
Private/ Health insurance	90 (29.1)	22 (19.5)	68 (34.5)	
Other	1 (0.3)	1 (0.9)	0 (0.0)	
**Mode of delivery**				0.073
Vaginal	164 (53.1)	67 (59.8)	97 (49.2)	
C-section	145 (46.9)	45 (40.2)	100 (50.8)	
**Child’s sex**				0.061
Female	132 (42.7)	40 (35.7)	92 (46.7)	
Male	177 (57.3)	72 (64.3)	105 (53.3)	
**Child’s skin color**				0.773
White	203 (65.7)	132 (66.7)	71 (63.4)	
Mixed race	89 (28.8)	54 (27.3)	35 (31.3)	
Black	17 (5.5)	11 (5.6)	6 (5.4)	
**Mother currently lives with partner**				0.474
Yes	220 (71.2)	77 (68.8)	143 (72.6)	
No	89 (28.8)	35 (31.3)	54 (27.4)	
**Family currently receives governmental financial aid**				0.100
Yes	87 (28.3)	38 (33.9)	49 (25.1)	
No	220 (71.7)	74 (66.1)	146 (74.9)	
**Maternal current employment status**				0.340
Yes	203 (65.9)	70 (62.5)	133 (67.9)	
No	105 (34.1)	42 (37.5)	63 (32.1)	
**Maternal current smoking status**				0.625
Yes	40 (14.3)	16 (15.7)	24 (13.6)	
No	239 (85.7)	86 (84.3)	153 (86.4)	
**Mother current alcohol uses**				0.789
Yes	84 (30.1)	32 (31.1)	52 (29.5)	
No	195 (69.9)	71 (68.9)	124 (70.5)	
**Child is under mental health follow-up**				0.013
Yes	36 (12.6)	20 (19.0)	16 (8.9)	
No	249 (87.4)	85 (81.0)	164 (91.1)	
**Child’s school type**				0.051
Philanthropic	21 (9.1)	10 (12.3)	11 (6.3)	
Private	29 (12.6)	4 (4.9)	25 (14.4)	
Public	180 (78.3)	68 (82.9)	112 (79.3)	

^**a**^Note: Differences refer to missing data.

**Fig 2:**
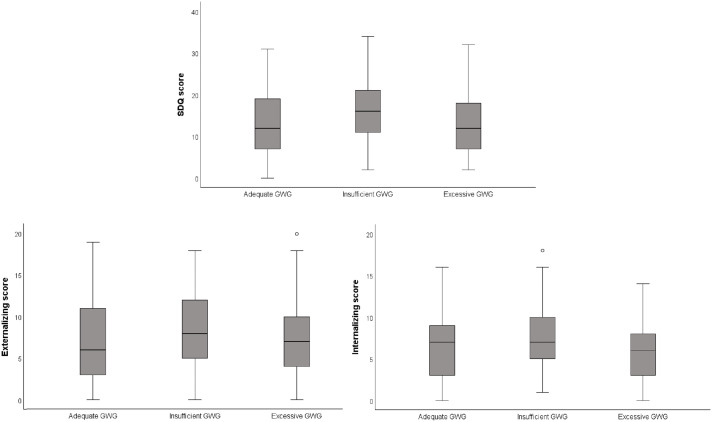
Children SDQ Score, Externalizing score and Internalizing score according to maternal gestational weight gain (GWG) adequacy. CLaB Study, Botucatu, Brazil, 2016-2024 (n = 309).

The median SDQ score among children of mothers with adequate GWG was 12.0 (IQR = 7–19), compared to 16.0 (IQR = 10.5–21.0) for those with insufficient gain and 12.0 (IQR = 7–18) for children of mothers with excessive GWG. These findings indicate significant differences in scores based on GWG adequacy (p = 0.016), specifically between excessive and insufficient GWG (p = 0.022). The difference between insufficient and adequate GWG approached statistical significance (p = 0.055), while no significant difference was observed between excessive and adequate GWG (p = 1.00). Variations in median scores for externalizing behaviors across GWG categories were not statistically significant (p = 0.105). However, there were significant differences in median internalizing scores (p = 0.006), particularly between the excessive GWG group (median = 6; IQR = 3–8) and the insufficient GWG group (median = 7; IQR = 5–10) (p = 0.004) (Fig 2).

Table 2 displays the results of the adjusted analyses between gestational weight gain (GWG) adequacy and the score indicative of behavioral problems in children, using two different reference categories: adequate GWG and excessive GWG. When adequate GWG was used as the reference category, the prevalence ratios varied but did not reach the threshold for statistical significance. However, when excessive GWG was used as the reference, children born to mothers with insufficient weight gain had a higher prevalence of these problems (PR = 1.48; 95% CI 1.02–2.13).

**Table 2:** Poisson regression between gestational weight gain adequacy and behavioral problems in childhood. CLaB Study, Botucatu, Brazil, 2016-2024 (n = 309).

Variables	Multivariate analysis	
	PR (95% CI)	p-value
**Gestational weight gain adequacy** ^#^		
Adequate	1.0	
Excessive	0.88 (0.60–1.28)	0.503
Insufficient	1.30 (0.89–1.89)	0.169
**Gestational weight gain adequacy** ^*^		
Adequate	1.14 (0.78–1.66)	0.503
Excessive	1.0	
Insufficient	1.48 (1.02–2.13)	0.037
**Child’s sex**		
Male	1.0	
Female	0.68 (0.48–0.95)	0.022
**Mother’s skin color**		
White/ yellow	1.0	
Mixed race	1.23 (0.91–1.67)	0.179
Black	0.63 (0.31–1.29)	0.206
**Mother lived with a partner**		
Yes	1.0	
No	1.21 (0.84 −1.75)	0.299
**Mother smoked during pregnancy**		
No	1.0	
Yes	1.44 (0.90–2.32)	0.129
**Maternal employment status**		
Yes	1.0	
No	1.09 (0.78–1.52)	0.624
**Pre-pregnancy nutritional status**		
Normal weight	1.0	
Underweight	1.22 (0.74–2.01)	0.446
Overweight	1.09 (0.74–1.60)	0.654
Obesity	1.06 (0.72–1.56)	0.772
**Mother’s education at Birth**		
≥ 12 years	1.0	
9–11 years	0.59 (0.42–0.83)	0.002
< 8 years	0.77 (0.47–1.27)	0.302
**Mother’s age**	0.98 (0.95–1.01)	0.121
**Parity**	1.02 (0.84–1.23)	0.858

# Adequate GWG as the reference category.

* Excessive GWG as the reference category.

The adequacy of GWG influenced the prevalence of internalizing problems. Multivariate analysis indicated that, compared to children whose mothers had adequate weight gain, children of women with excessive weight gain had fewer behavioral problems related to the internalizing domain (PR = 0.66; 95% CI = 0.47–0.94). An alternative analysis, using excessive GWG as the reference category, showed that both adequate and insufficient weight gain were associated with approximately a 50% increase in the prevalence of these problems. Regarding problems in the externalizing domain, no association was found with gestational weight gain adequacy (Table 3).

**Table 3:** Poisson regression between gestational weight gain adequacy and internalizing and externalizing behavioral problems in childhood. CLaB Study, Botucatu, Brazil, 2016-2024 (n = 309).

Variables	Internalizing problems		Externalizing problems	
	Multivariate analysis		Multivariate analysis	
	PR (95% CI)	p-value	PR (95% CI)	p-value
**Gestational weight gain adequacy** ^#^				
Adequate	1.0		1.0	
Excessive	0.66 (0.47–0.94)	0.020	0.78 (0.50–1.22)	0.276
Insufficient	1.03 (0.73–1.46)	0.877	1.07 (0.68–1.70)	0.769
**Gestational weight gain adequacy** ^*^				
Adequate	1.51 (1.07–2.13)	0.020	1.28 (0.82–2.00)	0.276
Excessive	1.0		1.0	
Insufficient	1.55 (1.07–2.23)	0.019	1.37 (0.84–2.24)	0.203
**Child’s sex**				
Male	1.0		1.0	
Female	0.84 (0.62–1.14)	0.259	0.62 (0.41–0.95)	0.028
**Mother’s skin color**				
White/ yellow	1.0		1.0	
Mixed race	1.03 (0.76–1.38)	0.862	1.17 (0.79–1.74)	0.441
Black	0.41 (0.18–0.92)	0.032	0.94 (0.44–1.97)	0.860
**Mother lived with a partner**				
Yes	1.0		1.0	
No	1.23 (0.85–1.76)	0.269	1.10 (0.67–1.79)	0.708
**Mother smoked during pregnancy**				
No	1.0		1.0	
Yes	0.96 (0.59–1.56)	0.868	1.63 (0.93–2.85)	0.087
**Maternal employment status**				
Yes	1.0		1.0	
No	1.22 (0.88–1.68)	0.230	1.10 (0.72–1.67)	0.666
**Pre-pregnancy nutritional status**				
Normal weight	1.0		1.0	
Underweight	0.91 (0.42–1.95)	0.801	1.19 (0.55–2.59)	0.659
Overweight	1.15 (0.79–1.68)	0.454	0.98 (0.59–1.62)	0.924
Obesity	1.23 (0.86–1.74)	0.255	1.32 (0.85–2.06)	0.222
**Mother’s education at Birth**				
≥ 12 years	1.0		1.0	
9–11 years	0.67 (0.47–0.94)	0.021	0.50 (0.32–0.78)	0.002
< 8 years	1.34 (0.88–2.03)	0.170	0.64 (0.34–1.22)	0.175
**Mother’s age**	0.98 (0.95–1.01)	0.130	0.98 (0.95–1.02)	0.301
**Parity**	0.98 (0.83–1.15)	0.790	0.97 (0.77–1.23)	0.824

# Adequate GWG as the reference category.

* Excessive GWG as the reference category.

## Discussion

Our hypothesis that gestational weight gain outside the ranges considered most favorable for maternal and child health could influence behavioral problems in 7- to 8-year-old children was only partially confirmed. Insufficient weight gain, only when compared to excessive weight gain, increased the prevalence of children with probable behavioral health disorders by 50%, independent of maternal pregestational nutritional status and various sociodemographic factors. However, compared to children whose mothers had adequate weight gain during pregnancy, the effect of insufficient weight gain did not reach statistical significance.

As mentioned in the introduction, the literature on the relationship between gestational weight gain and behavioral disorders in children is inconsistent. Studies in high-income countries such as the USA, Canada, Australia, Sweden, and China, aggregated in a meta-analysis, showed a small but significant adverse effect of excessive weight gain on neurodevelopmental and behavioral disorders in children and adolescents [5]. However, this finding was not observed in the Avon Longitudinal Study of Parents and Children (ALSPAC) [8], and inconsistencies were also found when evaluating the internalizing and externalizing domains: excessive weight gain in women with pregestational overweight was associated with higher scores in these two domains [9] (Tore) or with no such association [10].

Thus, a possible explanation for the divergence of our findings in relation to previous studies lies in the differences in living conditions between the populations studied.

It is worth noting that during the gestation period of the children in our cohort (2014–2016), the country was entering a recession, with rising unemployment and inflation, as well as periods of political instability. These conditions differ significantly from the stability and lower inequality found in the countries where most of the studies that detected the relationship between gestational weight gain and neurodevelopmental disorders were conducted.

Regardless of the reason for insufficient weight gain during pregnancy, it is likely associated with a low intake of essential micronutrients and macronutrients needed for fetal development. Maternal nutrition influences the early stages of embryo development, a critical period during which nutrient scarcity impacts the formation of brain structures, such as neuron proliferation and myelination [21,22]. Epigenetic modifications are also a potential mechanism by which low weight gain (poor nutrition) during pregnancy can influence children’s behavior at later ages [22].

Another finding of our study concerns the prevalence of a specific group of behavioral problems—those grouped within the internalizing domain of the SDQ. Among children of mothers with excessive weight gain, these problems were less frequent than in those whose mothers had adequate or insufficient weight gain. Conversely, the prevalence was higher when maternal weight gain was adequate or insufficient. To help interpret this result, we drew on the literature and formulated a hypothesis. The protective effect of excessive weight gain on internalizing problems could stem from this group of women experiencing fewer mental health issues. In the same locality, a previous study precisely identified that insufficient weight gain was associated with mental health problems, which were less common among pregnant women with excessive weight gain [23]. Supporting this hypothesis, a protective effect of better maternal mental health on child internalizing problems has already been documented in a meta-analysis including both cross-sectional and longitudinal studies [24]. It is also possible that residual confounding or other unmeasured factors, such as social support or access to healthcare [25], may have contributed to this apparent protective association.

The absence of an association between GWG and externalizing problems is consistent with findings from the Canadian APrON cohort [10] but contrasts with other international studies that have reported such an association [9,26]. It is also possible that externalizing behaviors, as well as internalizing ones, are more strongly influenced by other unmeasured factors — such as parenting style, exposure to violence, maternal depression or anxiety, or environmental toxicant exposure [25]— which were not assessed in our study.

Another finding that requires careful consideration is the high proportion of children identified as exhibiting behavioral problems. More than one-third of the children in our cohort scored 17 or higher on the SDQ, a value close to what was found in a population-based cohort in the Western Amazon region [27] and in other Brazilian studies [28–30], but higher than the 14% detected by another Brazilian study conducted in São Paulo [31]. The high prevalence of behavioral problems observed underscores the urgent need for systematic mental health screening in childhood. These difficulties, if not identified and managed early, may impair academic performance, peer relationships, and long-term wellbeing

Additionally, it is important to consider that data collection took place two to three years after the peak of the COVID-19 pandemic. In other words, the high prevalences observed cannot be fully attributed to the effects of the pandemic, but rather to conditions specific to the study context, as noted by other researchers [32,33].

The prevalence of internalizing problems was even higher, reaching 37.5% of the children, and one in four exhibited externalizing problems. These results are well above those reported for European children—18.4% for internalizing and 7.8% for externalizing problems [34] and for Spanish children aged 6–8 years, with 21.5% internalizing and 15.0% externalizing problems [35]. However, they are closer those observed in another Brazilian sample [36].

Some limitations should be noted, and their implications considered. The evaluation of emotional and behavioral problems in children was conducted at a single time point, at ages 7–8, and during the post-COVID-19 pandemic period, limiting the ability to analyze trajectories or behavioral changes throughout childhood. Despite the follow-up losses between the two phases of the study, the analysis maintained high statistical power. Moreover, the sociodemographic and obstetric characteristics of the mothers and children in the initial cohort were similar to those of the analyzed sample, preserving the representativeness of the study population. Differences in both the exposure variable and sociodemographic/obstetric characteristics were no greater than 4 percentage points between the full cohort and the final analytical sample.

In addition, pre-gestational weight was obtained from the medical records of the prenatal care service. Although this information is usually based on self-report and may be subject to measurement error, previous studies indicate that the magnitude of this error is generally small [37,38].

Additionally, the lack of data on maternal mental health, such as depression or anxiety, limited our ability to explore potential interactions between these factors: maternal mental health, gestational weight gain, and child behavior, as previously reported in the literature [7,39]. Finally, although the study was conducted in a single Brazilian city, with characteristics representative of many similar municipalities, the results may not be generalized to different cultural, economic, or healthcare settings.

On the other hand, the results of this study underscore the need for early and comprehensive interventions during pregnancy to improve maternal and child health. Future research should adopt more detailed longitudinal assessments, with multiple behavioral data collections, to better understand the association of GWG with trajectories of emotional and behavioral child development. Furthermore, the inclusion of maternal mental health information, such as depression and anxiety, could elucidate the interactions between psychosocial factors and gestational weight gain, allowing for the exploration of mediation or moderation hypotheses. Although children’s emotional and behavioral problems are known to be influenced by multiple factors, prenatal nutritional monitoring in healthcare settings should prioritize strategies that promote adequate gestational weight gain, considering pre-pregnancy nutritional status and the individual characteristics of each pregnant woman. Additionally, the inclusion of tools like the Strengths and Difficulties Questionnaire in primary healthcare, especially in vulnerable populations, can facilitate early interventions for children experiencing emotional and behavioral problems, improving their quality of life.
